# Editorial: Are machine learning, AI, and big data tools ready to be used for sustainable development? Challenges, and limitations of current approaches

**DOI:** 10.3389/fdata.2023.1301903

**Published:** 2023-11-21

**Authors:** Elisa Omodei, Dohyung Kim, Manuel Garcia-Herranz, Vedran Sekara

**Affiliations:** ^1^Department of Network and Data Science, Central European University, Vienna, Austria; ^2^UNICEF, New York, NY, United States; ^3^Department of Computer Science, IT University of Copenhagen, Copenhagen, Denmark

**Keywords:** biases and limitations of big datasets, data science for social good, data on vulnerable populations, computational social science, big data for development, AI for social good, machine learning for humanitarian work

The United Nations Sustainable Development Goals (SDGs) constitute a blueprint of the challenges society is facing today; from ending poverty, hunger, and gender inequalities, to combating climate change and building sustainable cities. To reach the 17 SDGs by 2030, governments and international organizations need to be able to monitor progress, estimate the impact of potential interventions, and make forecasts on how the situation is likely to evolve. Today, scientific communities, NGOs and international development agencies are extensively leveraging the potential of Big Data, Machine Learning (ML), and Artificial Intelligence (AI) tools to address these challenges. For instance, satellite imagery, mobile phone, and social media data have been used, in combination with different computational techniques, to estimate poverty, to predict population displacements in the aftermaths of a natural disaster, and to quantify the impact of human mobility during an epidemic outbreak. However, during our work in different UN agencies and academic institutions we have encountered multiple challenges and limitations to these new tools. It is a broad spectrum of issues, from difficulties of getting data, to more complex problems with data representativeness and bias, models not being robust, explainable, or transparent, to models which only focus on optimizing short-term goals, or rely on incomplete proxies. This assemblage of issues inspired the Research Topic. Our goals are to crowdsource knowledge on whether data-driven technologies are ready to be used for sustainable development, and to map out their limitations.

We got a large diversity of submissions focusing on different aspects, from data, and modeling, to governance, and meta studies. Below we summarize the six contributions. Water is essential for life, however, human-caused climate change has over the past decades globally affected both the quality and quantity of drinking water. Using the UK water infrastructure as use-case, Hazell et al. explore key challenges associated with using a data-driven approach for managing water systems. They identify different forms of challenges, from open dataset having more than 20 different formats while being under more than 30 types of licenses, to how water resources and systems are managed, and most importantly, that datafication and digitalization of water systems introduces new unknowns and complexities which are not trivial to foresee and plan.

Piaggesi et al. look into how novel digital traces can provide a complementary data source. Up-to-date and fine-grained measurements of socioeconomic indicators are vital in order to combat poverty. However, traditional poverty datasets are not regularly updated and can suffer from too coarse-grained spatial resolutions. Focusing on data collected from Facebook's advertising platform they model the socioeconomic distribution for four cities, selected from low-, middle-, and high-income countries. While they demonstrate that such data can be used to accurately map socioeconomic status, their key finding is that such models do not necessary generalize across urban regions. For instance, models calibrated on one city do not perform much better than random guessing for a different city. As such, they raise important questions on model generalizability.

Sartirano et al. also explore the advantages and challenges of using big data to derive relative wealth indices, with a focus on Indonesia. They compare the Relative Wealth Index (RWI), formulated through ML techniques, with the USAID's Demographic Health Survey (DHS) and the Indonesian National Socioeconomic Survey. Their methodology overcomes several limitations of traditional surveys, offering efficient data collection and extending the reach beyond areas not covered by the DHS. However, the authors stress the importance of leveraging diverse data sources and methodologies for well-informed decision-making and the need for regular updates in ML-based indices to reflect evolving societal profiles.

Pastor-Escuredo et al. delve into the intricacies of future societal systems, which they expect to be characterized by a mix of human behaviors and data-driven collective action. They propose a novel paradigm of multi-scale governance that underpins the Data Revolution in an increasingly digitalized world. The authors underscore the necessity for innovative forms of digital policy and governance, the pivotal role of AI in sustainable development, and the crucial need for coordination between top-down agencies and bottom-up digital platforms. This approach, the authors argue, will enable the implementation of data-driven policies that foster sustainable development based on collective intelligence.

Rocca et al. provide an overview of the opportunities that Natural Language Processing (NLP) is opening up for the humanitarian sector. Unstructured text data, such as interviews, news, and social media text, often encode relevant information for response planning and anticipatory action. However, despite its potential, the use of NLP in the humanitarian sector is still sporadic. The authors identify three main technical challenges behind this: the need for more domain-specific resources for training and benchmarking, the need for better multilingual technology, and the need for limiting the negative impact of model biases and for enhancing explainability. They hence urge the creation of a cross-functional humanitarian NLP community as the key to ensure impactful and ethical applications of NLP in humanitarian contexts.

Bratt et al. perform a meta study and use network analysis to analyze collaborations between scientists from the global north and global south (N-S), focusing on datasets submitted to the genetic sequence database GenBank. The authors find that collaborations between mixed income groups and Science & Technology capacity groups are infrequent, and, when they do occur, they are bursty, suggesting that these collaborations are formed and maintained only while reacting to infectious disease outbreaks such as Ebola or COVID-19. This shows how understanding the structures and dynamics of collaboration networks can help design interventions to support critical collaborations in future global health crises.

As these contributions highlights, there are lot of opportunities to use data-driven tools and technologies for public benefit, however, the challenges these new techniques bring are novel and many. Systemic changes need to be enacted for the world to benefit from these technologies. For instance, digitalizing a system will not necessary effectivize or improve the system, as digitalizing a broken process will leave you with a digitized, broken process. To ensure that these systems and models work for everyone, we need to ensure that science and research are not happening in seperate spaces (e.g., north and south, as Bratt et al. highlight). Similarly, the issues of low model transferability and bad generalizability could be addressed through the creation of domain-specific datasets. The tools are there, but it is not the tools that set the limits; currently it is the datasets, the applications, and the missing collaborations. These need to be addressed for ML, AI, and Big Data tools to be ready for sustainable development.

## Author contributions

EO: Writing – original draft, Writing – review & editing. DK: Writing – original draft, Writing – review & editing. MG-H: Writing – original draft, Writing – review & editing. VS: Writing – original draft, Writing – review & editing.

